# Spiroheterocyclization of Methyl 1-Aryl-3-cinnamoyl-4,5-dioxo-4,5-dihydro-1*H*-pyrrole-2-carboxylates by the Action of 3-(Arylamino)-1*H*-inden-1-ones

**DOI:** 10.3390/molecules171213787

**Published:** 2012-11-22

**Authors:** Pavel S. Silaichev, Valeriy O. Filimonov, Pavel A. Slepukhin, Andrey N. Maslivets

**Affiliations:** 1 Department of Organic Chemistry, Perm State National Research University, Perm 614066, Russia; Email: silaychev@yahoo.com (P.S.S.); filimondobryi@mail.ru (V.O.F.); 2 I. Ya. Postovskiy Institute of Organic Synthesis, Russian Academy of Science, Ural Branch, Yekaterinburg 620219, Russia; Email: slepukhin@ios.uran.ru

**Keywords:** pyrrole-2,3-diones, enamines, spiro[indeno[1,2-b]pyrrole-3,2'-pyrroles]

## Abstract

Methyl 1-aryl-3-cinnamoyl-4,5-dioxo-4,5-dihydro-1*H*-pyrrole-2-carboxylates interact with 3-(arylamino)-1*H*-inden-1-ones to give the corresponding 1,1'-diaryl-3'-cinnamoyl-4'-hydroxy-1*H*-spiro[indeno[1,2-b]pyrrole-3,2'-pyrrole]-2,4,5'(1'*H*)-triones in good yields.

## 1. Introduction

Spiro compounds represent an important class of naturally occurring substances characterised by their highly pronounced biological properties [1,2,3]. On the other hand, over the past three decades spiro compounds have received considerable attention owing to their diverse chemotherapeutic potential, including antineoplastic activities [4]. Some spiro compounds have been implemented as antimicrobial, antitumour and antibiotic agents [5,6]. In the arena of photochromism, spiro compounds, due to their steric constraints, equilibrate with the corresponding non-spiro analogue and exhibit various photochemical phenomena. Some related applications based on this equilibrium are self-development photography, actinometry, displays, filters and lenses of variable optical density, *etc*. [7,8,9].

A convenient approach to the synthesis of spirobisheterocycles is the interaction of 4-acyl-5-methoxycarbonyl-1*H*-pyrrole-2,3-diones with enamines. It was previously shown that methyl 3-acyl-1-aryl-4,5-dioxo-4,5-dihydro-1*H*-pyrrole-2-carboxylates react with acyclic [10], carbocyclic [11,12,13], and heterocyclic [14,15,16] enamines as 1,3-C,N-binucleophiles according to the scheme involving initial attack by the *β*-CH group in the enamino fragment on the C^5^ atom in the pyrrole ring and subsequent closure of new pyrrole ring via intramolecular attack by the NH group of enamines on the ester carbonyl carbon atom of dioxopyrroles and elimination of methanol. These reactions lead to the formation of spiro heterocyclic systems, such as 1,7-diazaspiro[4.4]nonane [10], spiro[indole-3,2'-pyrrole] [11,12,13], spiro[pyrrole-2,5'-pyrrolo[2,3-d]pyrimidine [14], and spiro[pyrrolo[2,1-a]iso-quinoline-2,2'-pyrrole] derivatives [15,16].

On the other hand, we found that 3-(arylamino)-1*H*-inden-1-ones react with ethyl 1-aryl-4,5-dioxo-2-phenyl-4,5-dihydro-1*H*-pyrrole-3-carboxylates as 1,5-CH,СH-binucleophiles according to the scheme which consists of an initial nucleophilic addition of an activated CH group *ortho* disposed toward an arylamino group of cyclic enamino ketones to the C^4^ carbon atom of pyrrolediones and the subsequent closure of the 1,4-dihydropyridine ring via intramolecular attack by the *β*-CH group of the enamino fragment onto the C^4^ atom in the pyrroledione ring. The last step of the transformation is accompanied by the elimination of a water molecule and is completed by the substituted spiro[indeno[1,2-b]quinoline-10,3'-pyrrole] derivative formation [17]. Reactions of five-membered carbocyclic enamino ketones with methyl 1-aryl-3-cinnamoyl-4,5-dioxo-4,5-dihydro-1*H*-pyrrole-2-carboxylates have not been described previously. We report herein another type of spirohetero-cyclization of substituted 1*H*-pyrrole-2,3-dione derivatives under the action of these enamino ketones, which act here as 1,3-CH,NH-binucleophiles.

## 2. Results and Discussion

**Scheme 1:**
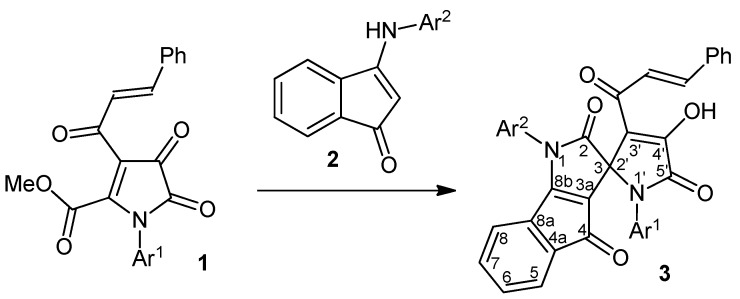
Synthesis of spiro[indeno[1,2-b]pyrrole-3,2'-pyrroles] **3**.

During the course of our studies on nucleophilic transformations of monocyclic 1*H*-pyrrole-2,3-diones under the action of bifunctional nucleophiles, we have examined an interaction between methyl 1-aryl-3-cinnamoyl-4,5-dioxo-4,5-dihydro-1*H*-pyrrole-2-carboxylates **1** and 3-(arylamino)-1*H*-inden-1-ones **2**. As a result we have found that refluxing of **1** and **2** taken in a 1:1 molar ratio in dry toluene for 5–6 h under TLC monitoring provides the corresponding 1,1'-diaryl-3'-cinnamoyl-4'-hydroxy-1*H*-spiro[indeno[1,2-b]pyrrole-3,2'-pyrrole]-2,4,5'(1'*H*)-triones **3** in good yields (Scheme 1, Table 1).

**Table 1:** Synthesis of spiro[indeno[1,2-b]pyrrole-3,2'-pyrroles] **3**.

Entry	Ar^1^	Ar^2^	Product 3	Yield (%)
1	Ph	4-BrC_6_H_4_	3a	80
2	4-MeC_6_H_4_	Ph	3b	82
3	4-MeC_6_H_4_	4-MeC_6_H_4_	3c	79
4	4-MeC_6_H_4_	4-MeOC_6_H_4_	3d	84
5	4-MeOC_6_H_4_	Ph	3e	81
6	4-MeOC_6_H_4_	4-MeC_6_H_4_	3f	80
7	4-MeOC_6_H_4_	4-MeOC_6_H_4_	3g	79
8	4-MeOC_6_H_4_	4-BrC_6_H_4_	3h	83

**Figure 1:**
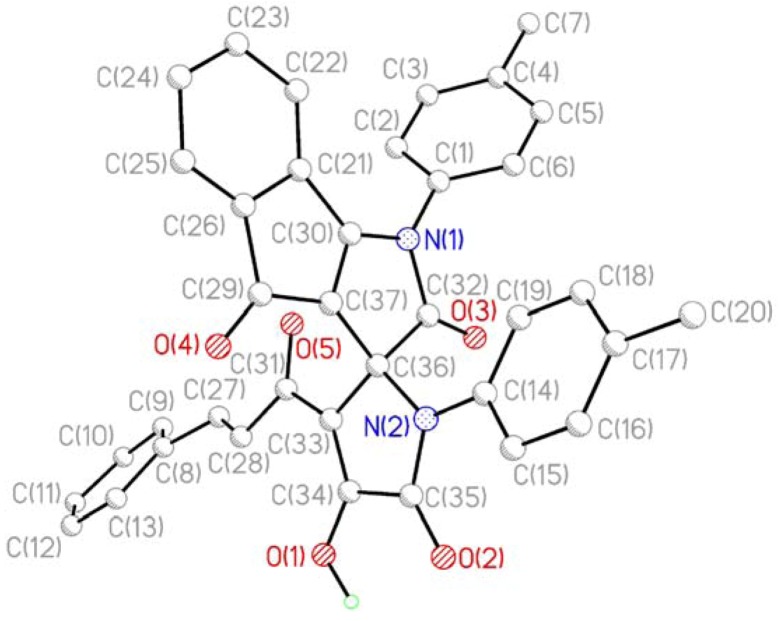
X-ray structure of the compound **3c**.

**Figure 2:**
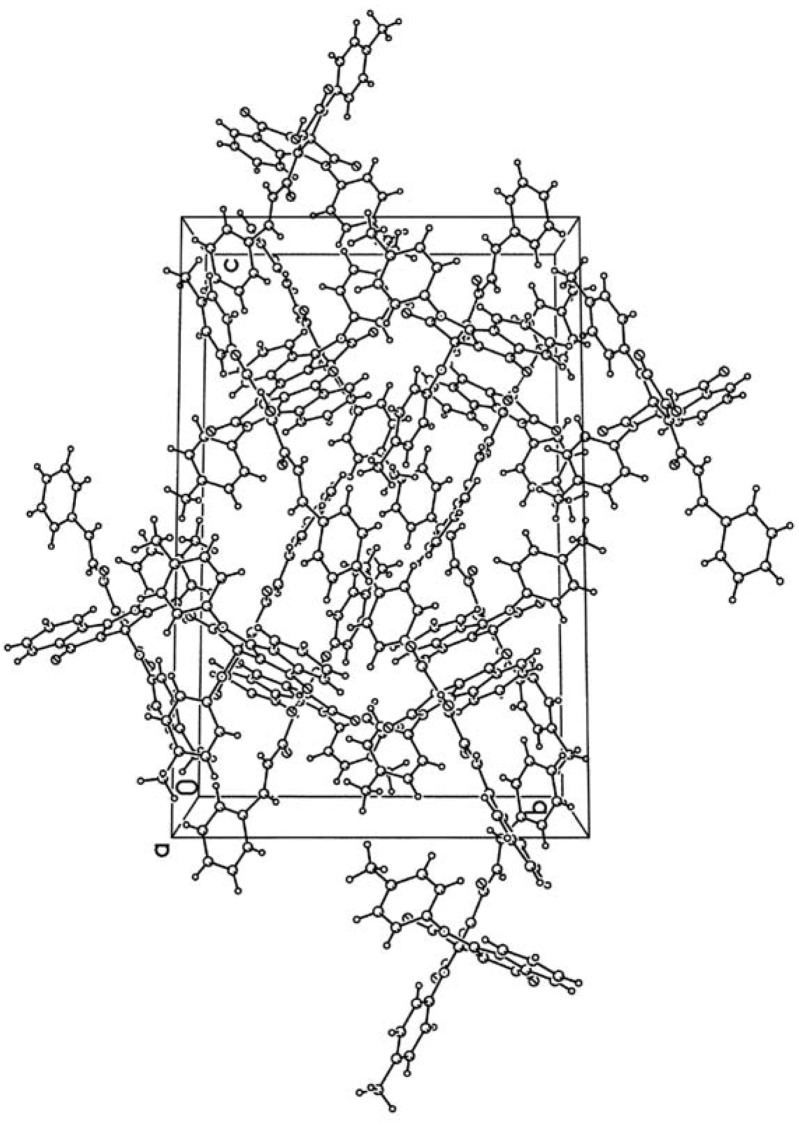
The figure of crystal packing of the compound **3c**.

Compounds **3** are red crystal substances readily soluble in DMSO and DMF, poorly soluble in other common organic solvents, and insoluble in saturated hydrocarbons and water. The products give a positive test (cherry-red coloration) with iron(III) chloride for the presence of enol hydroxyl groups. The IR spectra of **3** have absorption bands inherent to stretching vibrations of the enolic hydroxy group (3161–3188 cm^−1^, broadened band), two lactam carbonyl groups C^5'^=O (1763–1781 cm^−1^) and C^2^=O (1715–1732 cm^−1^), and two ketone carbonyl moieties C^4^=O (1667–1680 cm^−1^) and C^3'^-C=O (1640–1647 cm^−1^). ^1^H-NMR spectra of **3** display signals of protons in the aromatic rings and substituents attached thereto, two doublets from the protons of the ethylene fragment of the cinnamoyl substituent (δ 7.64–7.66 and 7.72–7.75 ppm) with coupling constant (^3^*J*) values of about 16 Hz, and a broadened singlet from the enolic hydroxy proton (δ 13.31–13.45 ppm). In the ^13^C-NMR spectra of **3d** we have observed carbon atom signals of the aromatic and aliphatic fragments, the carbonyl carbon atom of the cinnamoyl moiety (δ 182.46 ppm), ketone carbonyl carbon atom C^4^ (δ 183.74 ppm), lactam carbonyl carbon atoms C^2^ (δ 171.89 ppm) and C^5'^ (δ 165.38 ppm), and spiro carbon atom (δ 68.34 ppm). The structure of **3c** was unambiguously confirmed by single-crystal X-ray crystallography (Figure 1 and Figure 2).

According to the XRD study two independent molecules of compound **3c** are crystallized in the centrosymmetric space group P2_1_/n of monoclinic crystal system in the solvated form. Due to thermal disordering solvate atoms are not positioned with good precision and the SQUEEZE procedure in the PLATON program [18] was applied for its treatment. A general view of one independent molecule is presented on Figure 1; the second molecule has similar geometry and numeration of its atoms has an additional index “A”.

Bond lengths and bond angles in compound **3c** are close to standard values. The measured angle between the planes of the heterocycles at the spiro-node amounts to 89.5 deg. The most significant feature of the crystal packing is the formation of dimers at the expense of intermolecular HB O1-H1-O4A [O1-H1 1.05(3), H1-O4A 1.71(2), O1-O4A 2.644(3) Å, angle O1H1O4A 144(1)°] and O1A-H1A-O4 [O1A-H1A 0.71(3), H1A-O4 1.98(3), O1A-O4 2.622(3) Å, angle O1AH1AO4 151(1)°].

**Scheme 2:**
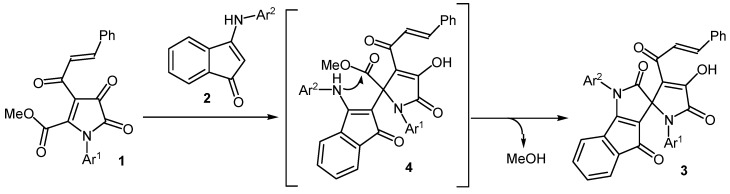
The proposed mechanism for the synthesis of spiro[indeno[1,2-b]pyrrole-3,2'-pyrroles] **3**.

The formation of compounds **3** occurs due to the addition of the *β*-CH group of the enamino fragment of indenones **2** to the atom C^5^ of pyrrolediones **1**, followed by the closure of the pyrrole ring through the intramolecular attack of the amino group of indenones **2** on the ester carbonyl group in the position *5* of the pyrroledione with simultaneous elimination of methanol (Scheme 2). Our attempts to isolate intermediates **4** of this transformation failed. Presumably, the rate of intramolecular cyclization compounds **4** higher than the rate of their formation.

## 3. Experimental

### 3.1. General

Melting points were recorded on a Gallenkamp apparatus. IR spectra (mineral oil) were recorded on an FMS-1201 spectrophotometer. The ^1^H- and ^13^C-NMR spectra were recorded on a Bruker AM 400 spectrometer (at 400 MHz for ^1^H-NMR and 100 MHz for ^13^C-NMR) with DMSO-*d_6_* as solvent and TMS as internal reference, chemical shifts are expressed as δ ppm. All reactions were followed by TLC (silica gel, aluminum sheets, Silufol, 5:1, benzene-ethyl acetate). X-Ray analyses were performed using an “Xcalibur 3” diffractometer.

CCDC 909 605 contains the supplementary crystallographic data for compound **3c**. These data can be obtained free of charge via www.ccdc.cam.ac.uk/conts/retrieving.html (or from the CCDC, 12 Union Road, Cambridge CB2 1EZ, UK; fax: +44-1223-336033; e-mail: deposit@ccdc.cam.ac.uk).

### 3.2. General Procedure for Preparation of Spiro[indeno[1,2-b]pyrrole-3,2'-pyrroles] ***3a–h***

A solution of 1.0 mmol of pyrroledione **1** and enamine **2** in dry toluene (20 mL) was heated under reflux for 5–6 h (progress of the reactions was monitored using TLC with 5:1 benzene-ethyl acetate as eluent). The mixture was then cooled, the resulted precipitate was filtered off and recrystallized from toluene.

*1-Bromo-3'-cinnamoyl-4'-hydroxy-1'-phenyl-1H-spiro[indeno[1,2-b]pyrrole-3,2'-pyrrole]-2,4,5'(1'H)-trione* (**3a**): Red solid, yield 80%, mp 272–274 °C. IR ν_max_: 3173 (OH), 1768 (C2=O), 1726 (C5'=O), 1671 (C4=O), 1642 (C3'-C=O) cm^−1^. ^1^H-NMR δ: 6.51 (d, 1H, H-8, *J* = 7.3 Hz), 7.13–7.89 (m, 18H, H-arom), 7.66 (d, 1H, COCH=CHPh, *J* = 15.8 Hz), 7.75 (d, 1H, COCH=CHPh, *J* = 15.8 Hz), 13.35 (s, 1H, OH).

*3'-Cinnamoyl-4'-hydroxy-1'-(4-methylphenyl)-1-phenyl-1H-spiro[indeno[1,2-b]pyrrole-3,2'-pyrrole]-2,4,5'(1'H)-trione* (**3b**): Red solid, yield 82%, mp 274–275 °C. IR ν_max_: 3174 (OH), 1781 (C2=O), 1725 (C5'=O), 1674 (C4=O), 1642 (C3'-C=O) cm^−1^. ^1^H-NMR δ: 2.29 (s, 3H, Me), 6.38 (d, 1H, H-8, *J* = 7.2 Hz), 7.02–7.69 (m, 17H, H-arom), 7.65 (d, 1H, COCH=CHPh, *J* = 16.1 Hz), 7.74 (d, 1H, COCH=CHPh, *J* = 16.1 Hz), 13.40 (s, 1H, OH).

*3'-Cinnamoyl-4'-hydroxy-1,1'-di(4-methylphenyl)-1H-spiro[indeno[1,2-b]pyrrole-3,2'-pyrrole]-2,4,5'(1'H)-trione* (**3c**): Red solid, yield 79%, mp 282–283 °C. IR ν_max_: 3174 (OH), 1763 (C2=O), 1727 (C5'=O), 1678 (C4=O), 1647 (C3'-C=O) cm^−1^. ^1^H-NMR δ: 2.28 (s, 3H, Me), 2.43 (s, 3H, Me), 6.39 (d, 1H, H-8, *J* = 7.3 Hz), 7.01–7.69 (m, 16H, H-arom), 7.64 (d, 1H, COCH=CHPh, *J* = 15.8 Hz), 7.74 (d, 1H, COCH=CHPh, *J* = 15.8 Hz), 13.31 (s, 1H, OH). The structure of compound **3c** was determined on a single-crystal X-ray diffractometer “Xcalibur 3” equipped with a CCD detector using the standard procedure (graphite-monochromated Mo*К_α_* radiation, Т = 295(2) К, ω-scanning, scanning step 1°). The red crystals had dimensions 0.25 × 0.20 × 0.15 mm, crystal system monoclinic, the space group P2_1_/n, unit cell parameters: *a* = 15.2533(6), *b* = 16.6750(12), *c* = 26.0000(19) Å, β = 101.496(4)°, *V* = 6480.4(7) Å^3^, Z = 8, *d_calc_* = 1.186 g/cm^3^. 23,715 reflections were collected in the range of angles of 2.57° < θ < 26.40°, from which 13,082 independent reflections (R_int_ = 0.0361) and 5076 with I > 2σ(I). The completeness of the experiment at the corners of θ ≤ 26.0° is 98.8%. The structure was solved by direct methods and refined by full-matrix least-squares procedure on F^2^ with the SHELXTL-97 [19] program in the anisotropic approximation for non-hydrogen atoms. Correction for absorption was not introduced (μ = 0.079 mm^−1^). The final refinement parameters: R_1_ = 0.0456, wR_2_ = 0.1037 for reflections with I > 2σ (I); R_1_ = 0.1174, wR_2_ = 0.1085 for all reflections, GooF = 1.019. Largest diff. peak and hole 0.186 and −0.240 ēÅ^−3^. Protons of NH-and OH-groups were located on the peaks of the spatial electron density and refined independently. The positions of the remaining H atoms were calculated geometrically and included in the refinement with the “riding model”. Disordered solvate was treated by the SQUEEZE procedure in the PLATON program [18].

*3'-Cinnamoyl-4'-hydroxy-1-(4-methoxyphenyl)-1'-(4-methylphenyl)-1H-spiro[indeno[1,2-b]pyrrole-3,2'-pyrrole]-2,4,5'(1'H)-trione* (**3d**): Red solid, yield 84%, mp 275–276 °C. IR ν_max_: 3180 (OH), 1777 (C2=O), 1732 (C5'=O), 1680 (C4=O), 1640 (C3'-C=O) cm^−1^; ^1^H-NMR δ: 2.29 (s, 3H, Me), 3.87 (s, 3H, OMe), 6.40 (d, 1H, H-8, *J* = 7.5 Hz), 7.02–7.69 (m, 16H, H-arom), 7.65 (d, 1H, COCH=CHPh, *J* = 15.7 Hz), 7.74 (d, 1H, COCH=CHPh, *J* = 15.7 Hz), 13.35 (s, 1H, OH). ^13^C-NMR δ ppm: 20.54 (Me), 55.47 (OMe), 68.34 (C-3), 108.08 (C-3a), 115.03 (C_m_Ar^2^), 117.72 (C-3'), 120.52–138.40, 142.74 (CO-CH=CH-Ph), 155.63 (C-8b), 159.89 (C_ip_Ar^2^), 165.38 (C-5'), 171.89 (C-2), 177.22 (C-4'), 182.46 (C3'-C=O), 183.74 (C-4).

*3'-Cinnamoyl-4'-hydroxy-1'-(4-methoxyphenyl)-1-phenyl-1H-spiro[indeno[1,2-b]pyrrole-3,2'-pyrrole]-2,4,5'(1'H)-trione* (**3e**): Red solid, yield 81%, mp 269–270 °C. IR ν_max_: 3180 (OH), 1773 (C2=O), 1715 (C5'=O), 1669 (C4=O), 1640 (C3'-C=O) cm^−1^. ^1^H-NMR δ: 3.74 (s, 3H, OMe), 6.38 (d, 1H, H-8, *J* = 7.5 Hz), 7.36–7.69 (m, 17H, H-arom), 7.65 (d, 1H, COCH=CHPh, *J* = 16.0 Hz), 7.73 (d, 1H, COCH=CHPh, *J* = 16.0 Hz), 13.40 (s, 1H, OH).

*3'-Cinnamoyl-4'-hydroxy-1'-(4-methoxyphenyl)-1-(4-methylphenyl)-1H-spiro[indeno[1,2-b]pyrrole-3,2'-pyrrole]-2,4,5'(1'H)-trione* (**3f**): Red solid, yield 80%, mp 252–253 °C. IR ν_max_: 3161 (OH), 1773 (C2=O), 1725 (C5'=O), 1671 (C4=O), 1644 (C3'-C=O) cm^−1^. ^1^H-NMR δ: 2.43 (s, 3H, Me), 3.74 (s, 3H, OMe), 6.39 (d, 1H, H-8, *J* = 7.3 Hz), 7.03–7.69 (m, 16H, H-arom), 7.65 (d, 1H, COCH=CHPh, *J* = 16.2 Hz), 7.73 (d, 1H, COCH=CHPh, *J *= 16.2 Hz), 13.35 (s, 1H, OH).

*3'-Cinnamoyl-4'-hydroxy-1,1'-di(4-methoxyphenyl)-1H-spiro[indeno[1,2-b]pyrrole-3,2'-pyrrole]-2,4,5'(1'H)-trione* (**3g**): Red solid, yield 79%, mp 259–260 °C. IR ν_max_ 3161 (OH), 1773 (C2=O), 1717 (C5'=O), 1667 (C4=O), 1642 (C3'-C=O) cm^−1^. ^1^H-NMR δ: 3.74 (s, 3H, OMe), 3.86 (s, 3H, OMe), 6.40 (d, 1H, H-8, *J* = 7.6 Hz), 7.03–7.73 (m, 16H, H-arom), 7.65 (d, 1H, COCH=CHPh, *J* = 15.7 Hz), 7.73 (d, 1H, COCH=CHPh, *J* = 15.7 Hz), 13.30 (s, 1H, OH).

*1-Bromo-3'-cinnamoyl-4'-hydroxy-1'-(4-methoxyphenyl)-1H-spiro[indeno[1,2-b]pyrrole-3,2'-pyrrole]-2,4,5'(1'H)-trione* (**3h**): Red solid, yield 83%, mp 256–257 °C. IR ν_max_ 3188 (OH), 1777 (C2=O), 1732 (C5'=O), 1673 (C4=O), 1644 (C3'-C=O) cm^−1^. ^1^H-NMR δ: 3.74 (s, 3H, OMe), 6.51 (d, 1H, H-8, *J* = 7.1 Hz), 7.02–7.87 (m, 16H, H-arom), 7.64 (d, 1H, COCH=CHPh, *J* = 16.0 Hz), 7.72 (d, 1H, COCH=CHPh, *J* = 16.0 Hz), 13.45 (s, 1H, OH).

## 4. Conclusions

The described reaction presents a rare example of a regioselective synthesis of a hardly accessible spiroheterocyclic system—spiro[indeno[1,2-b]pyrrole-3,2'-pyrrole] including various functional substituents in both heterocyclic fragments. Overall, we have succeeded in developing a method for synthesis of new functionalized spiro[indeno[1,2-b]pyrrole-3,2'-pyrroles derivatives of potential synthetic and pharmacological interest from the reaction of 1*H*-pyrrole-2,3-diones with 3-(arylamino)-1*H*-inden-1-ones. Our work presents a very simple reaction performed under neutral conditions and in the absence of any catalyst. From a structural viewpoint, the products are polycarbonyl compounds suitable for further elaboration. High yields and simple reaction and purification procedures are the key advantages of this approach.
